# Expression and prognostic value of AIM1L in esophageal squamous cell carcinoma

**DOI:** 10.1097/MD.0000000000034677

**Published:** 2023-08-25

**Authors:** Lu Zhou, Lanlan Gan, Zongwen Liu

**Affiliations:** a Second Affiliated Hospital of Zhengzhou University, Zhengzhou, Henan, China.

**Keywords:** AIM1L, esophageal squamous cell carcinoma, prognosis

## Abstract

**Background:**

Absent in melanoma 1-like (AIM1L), also known as crystalline beta gamma domain containing 2. The relationship between AIM1L and tumors has not been fully investigated, and the biological function of AIM1L in different tumors is unknown, so we bioinformatically explored a possible relationship between AIM1L and esophageal squamous cell carcinoma (ESCC).

**Methods:**

AIM1L mRNA expression was detected by the Gene Expression Omnibus database (GSE20347, GSE161533, and GSE53625), and protein level expression was detected by immunohistochemistry. The correlation between AIM1L expression and clinical pathological characteristics was evaluated by the Wilcoxon signed rank test or chi-square test. Kaplan–Meier analysis and Cox proportional risk regression model were used to determine the prognostic value of AIM1L in ESCC patients and establish and verify a nomogram. Find genes highly related to the expression of AIM1L, conduct GO and Kyoto encyclopedia of genes and genomes (KEGG) enrichment analysis, and conduct GSEA analysis on the gene set. The “CIBERSORT” R package was used to explore the relationship between AIM1L and immune infiltration, and the “OncoPredict” R package was used to explore the relationship between AIM1L and drug sensitivity.

**Results:**

Compared with the matched adjacent non-cancer tissues, the expression of AIM1L was down-regulated in ESCC tissues, and correlated with tumor grade. Kaplan–Meier survival analysis and Cox analysis showed that the low expression of AIM1L was related to the poor prognosis of ESCC patients. Enrichment analysis explained the possible function of AIM1L, GSEA determined the highly correlated signal pathway of AIM1L low expression phenotype, immune infiltration analysis determined that AIM1L was related to activated NK cells and macrophage M2, and drug sensitivity analysis determined that the low expression of AIM1L might be related to EGFR targeted drug resistance.

**Conclusion:**

AIM1L may be a candidate tumor suppressor gene for ESCC and an independent molecular biomarker for the prognosis of ESCC patients.

## 1. Introduction

Esophageal squamous cell carcinoma (ESCC) is one of the most common malignant tumors of the human digestive system. In 2020, there were about 604,000 new cases of esophageal cancer (ESCA) worldwide, accounting for 3.1% of the 36 cancer tumors, ranking 10th, and 544,000 deaths accounting for 5.5% of the 36 tumors, ranking 6th.^[1]^ Squamous cell carcinoma accounts for approximately 90% of ESCA cases worldwide and is the major histological type of ESCA.^[2]^ ESCC is not very common in Western countries, but it remains common in Asia (endemic areas) and Eastern Europe.^[3]^ Despite great improvements in the treatment techniques for ESCC, the prognosis of ESCC patients remains poor, with 5-year overall survival (OS) rates ranging from 26.2% to 49.4% after surgery.^[4]^ Therefore, identifying potential biomarkers of ESCC is of great significance for early diagnosis and prognosis prediction.

Absent in melanoma 1-like (AIM1L), also known as crystalline beta gamma domain containing 2. By exploring AIM1L in the National Center for Biotechnology Information (https://www.ncbi.nlm.nih.gov/), it was found that it was highly expressed in normal human tissues such as the esophagus and skin.^[5]^ A mass spectrometry analysis showed that AIM1L mRNA was highly expressed in some cancer cell lines, including breast cancer, colon cancer, ovarian cancer, and in noncancerous tissues only in the placenta. Esophagus and esophageal-related cancers were not involved in this study.^[6]^ A pharmacogenomic study showed that single nucleotide polymorphisms of AIM1L were associated with nausea and vomiting in cancer patients receiving opioid therapy.^[7]^ Bioinformatics analysis suggested that AIM1L expression is higher in liver tumors compared to normal liver tissue, and hepatocellular carcinoma patients with high AIM1L expression generally have shorter OS. Functional enrichment analysis suggested AIM1L may be involved in cell proliferation and migration.^[8]^ At present, there are few studies on AIM1L, and the relationship between AIM1L and most tumors, including ESCC, has not been reported. Therefore, it is urgent to study the related role of AIM1L in tumors including ESCC.

In this study, we first downloaded some gene expression datasets about ESCC from the Gene Expression Omnibus database and analyzed the expression of AIM1L among them, next, we explored the association of AIM1L with ESCC diagnosis and prognosis, we also analyzed whether AIM1L was associated with ESCC clinical features. After that, we constructed and validated a nomogram to predict the possible survival time of ESCC patients. To explore the possible role of AIM1L in ESCC, we identified genes that are highly correlated with AIM1L in ESCC, and then performed gene ontology (GO) and Kyoto encyclopedia of genes and genomes (KEGG) enrichment analysis of these genes. In addition, we also used gene set enrichment analysis (GSEA) to explore the functions and pathways enriched by AIM1L low expression group genes in ESCC. Using “CIBERSORT,” we found that AIM1L in ESCC is associated with an immune cell infiltration imbalance. Finally, the sensitivity of AIM1L and ESCC common drugs was analyzed. Our results showed that AIM1L is a promising molecular marker and therapeutic target of ESCC.

## 2. Materials and methods

### 2.1. Data collection and specimens

The gene expression datasets (GSE20347, GSE161533, and GSE53625) analyzed in this study are all from the Gene Expression Omnibus database (https://www.ncbi.nlm.nih.gov/geo/).^[9]^ GSE53625 is based on the GPL18109 platform and contains 179 pairs of mRNA expression information of ESCC and adjacent normal tissues. GSE20347 is based on the GPL571 platform, which is composed of 17 matched ESCC and normal tissues. GSE161533 is based on the GPL570 platform, which is composed of 28 matched ESCC, para tumor tissues, and normal tissues. GSE53625 contains comprehensive clinical information. Before analyzing the data from these datasets, we preprocessed the raw data of mRNA expression in all datasets. First, we averaged the expression data of the same gene in each dataset. Second, we normalized it using the “limma” package. It is worth noting that the platform used by GSE53625 is not marked with gene symbols, we used the documents published in the study of Shervin Alaei et al for gene ID conversion.^[10]^ In addition, we performed a log transform on the data for GSE161533. This study was analyzed using R software (v4.2.1).^[11]^ In addition, we collected paraffin sections of cancerous tissue and normal tissue adjacent to cancer from 2022 to 2023 from the Pathology Department of the Second Affiliated Hospital of Zhengzhou University. Inclusion criteria: all patients had specimens left in the Pathology Department of the Second Affiliated Hospital of Zhengzhou University; All patients were esophageal squamous cell carcinoma patients, aged from 18 to 75 years old, and all biopsy specimens were taken before anti-tumor treatment; All patients have corresponding informed consent. Exclusion criteria: the concurrent presence of other malignant tumors; Psychologically abnormal; There are contraindications of anti-tumor treatment. This experiment was approved by the Second Affiliated Ethics Committee of Zhengzhou University.

### 2.2. Expression analysis of AIM1L in multiple datasets

To explore the expression of AIM1L in ESCC, the “limma” package was used to analyze AIM1L expression in tumor and normal tissues among the 3 datasets, and paired t-tests were used to analyze whether there was a significant difference. Gene Expression Profile Interactive Analysis (GEPIA, http://gepia.cancer-pku.cn/), which can analyze the expression data of multiple genes in tumors in The Cancer Genome Altas database and the genotype-tissue expression database, is a powerful bioinformatics analysis tool.^[12]^ So we also utilized GEPIA to explore the expression amount of AIM1L in ESCA and normal esophageal tissues in The Cancer Genome Altas and GTEx.

### 2.3. Analysis of the diagnostic and prognostic value of AIM1L in ESCC

We used clinical data from GSE53625 for the next analyses. We divided ESCC patients into high and low-expression groups according to the median expression of AIM1L, performed Kaplan–Meier survival analysis by the “surminer” and “survival” packages, and constructed OS curves between the 2 groups to evaluate whether AIM1L was associated with OS. Then, by using the “survival” package, univariate and multivariate Cox regression proportional analysis was performed to determine whether AIM1L was an independent prognostic factor. Furthermore, to evaluate the value of AIM1L in ESCC diagnosis, we analyzed the receiver operating characteristic (ROC) curve and calculated the area under the curve using the “pROC” package.

### 2.4. Correlation analysis between AIM1L and clinical characteristics

To explore whether AIM1L expression levels are associated with clinical features of ESCC, we used the Wilcoxon signed rank test or chi-square test to analyze the correlation of AIM1L levels with the clinical information included in GSE53625, the R packages “complexheatmap” and “ggpubr” were used at this step.

### 2.5. Establishment and validation of a clinical prognostic model

To explore whether AIM1L together with clinicopathological features could help predict patient prognosis, based on GSE53625 AIM1L expression, age, tumor location, grade, T, N, and TNM stage, we constructed a nomogram and generated a calibration curve to verify reliability. This step used the R packages “rms,” “regplot,” and “survival.”

### 2.6. Identification of relevant genes for AIM1L and enrichment analysis

First, based on the gene expression information in the GSE53625 tumor samples, we performed a correlation analysis to probe for genes potentially associated with AIM1L in ESCC, setting the threshold value of | cor | > 0.5, *P* < .05. The genes that are highly related to AIM1L were illustrated by the Circos diagram. Next, we conducted GO and KEGG enrichment analysis on the acquired AIM1L-related genes to find out the possible functional mechanism and pathway of enrichment of these genes.^[13,14]^ In addition, we used GSEA (https://www.gsea-msigdb.org/gsea/index.jsp) to explore the pathway and functional mechanism of differential gene enrichment in the AIM1L low expression group.^[15]^ In this process, the R packages “ggplot2,” “ggpubr,” “ggExtra,” “circle,” “corrlot,” “org. Hs. e.g. db,” “enrichplot,” “clusterProfiler,” “circle,” “RColorBrewer,” “dplyr,” and “ComplexHeatmap” were applied.

### 2.7. Correlation analysis between AIM1L and immune cell infiltration in ESCC

We calculated the proportion of immune cells in GSE53625 tumor samples using the “CIBERSORT” package, which includes 22 immune cell subtypes.^[16]^ Then the differences in the infiltration levels of immune cells between the 2 groups with high and low expression of AIM1L were assessed, with a boxplot displaying the results. AIM1L was later analyzed for correlation with immune cells and represented with a lollipop plot. In addition, we also analyzed the correlation of AIM1L with immune checkpoint-related genes. In this step, “reshape2,” “ggpubr,” “violot,” “ggplot2,” “corrplot,” and “ggExtra” packages were used.

### 2.8. Sensitivity analysis of AIM1L with anticancer drugs

Genomics of Drug Sensitivity in Cancer (GDSC; https://www.cancerrxgene.org/) contains a large amount of cancer cell drug response molecular markers and drug sensitivity information, which focuses on identifying molecular biomarkers of drug sensitivity.^[17]^ To predict the potential impact of AIM1L on drug treatment of ESCC patients, we downloaded data from GDSC and analyzed the relationship between AIM1L and sensitivity to common chemotherapeutic and molecularly targeted agents in ESCC patients, including paclitaxel, cisplatin, oxaliplatin, 5-fluorouracil, irinotecan, docetaxel, gefitinib, erlotinib. In this step, “parallel,” “ggpubr,” “ggplot2,” and “oncoPredict” packages of R software were used.^[18]^

### 2.9. Immunohistochemical staining

Firstly, a 3 µm thick section was cut from paraffin-embedded tissue, dewaxed, and subjected to routine hydration, followed by antigen repair with citrate. After blocking with endogenous peroxidase, the antigen was incubated with AIM1L antibody (1:150, Novus, NBP1-90617) at room temperature for 30 minutes, followed by subsequent addition of post primary and polymer. The fresh DAB was moderately dyed, Haematoxylin was redyed, and the blue returning solution was dripped, and dehydrated by gradient ethanol, and the neutral resin was sealed. At 200 × Observe the slices under a light microscope and take photos based on the staining intensity and proportion of positive cells. The immunohistochemical staining results were determined by a semi-quantitative scoring method. Staining degree: negative staining 0 point, light yellow 1 point, brown yellow 2 points, brown 3 points. Positive range: 0 point for <5%, 1 point for 5% to 24%, 2 points for 25% to 50%, 3 points for 51% to 74%, and 4 points for ≥75%. Multiply the above 2 scores as the final score result: 0 is negative, <5 is low expression, and ≥5 is high expression.

## 3. Results

### 3.1. AIM1L mRNA expression in ESCC

**Figure 1.**
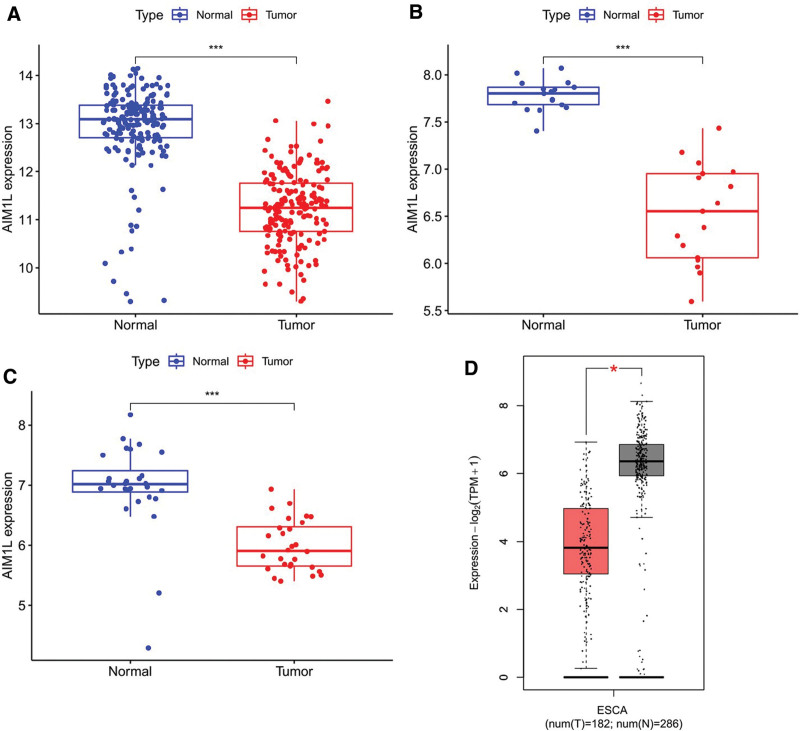
AIM1L expression in ESCC patients. (A) GSE53625, (B) GSE20347, (C) GSE161533, (D) expression of AIM1L in TCGA and GTEx data using GEPIA. Red represents the tumor group, gray represents the normal group. *P < .05, **P < .01, ***P < .05. AIM1L = absent in melanoma 1-like, ESCC = esophageal squamous cell carcinoma, GEPIA = gene expression profile interactive analysis, GTEx = genotype-tissue expression, TCGA = The Cancer Genome Altas.

In 3 datasets, GSE53625, GSE20347, and GSE161533, AIM1L mRNA expression is lower in ESCC tumor tissues compared to normal tissues (Fig. 1A–C). GEPIA analysis also revealed that AIM1L was downregulated in tumor tissues (Fig. 1D).

### 3.2. The diagnostic and prognostic value of AIM1L in ESCC

**Figure 2.**
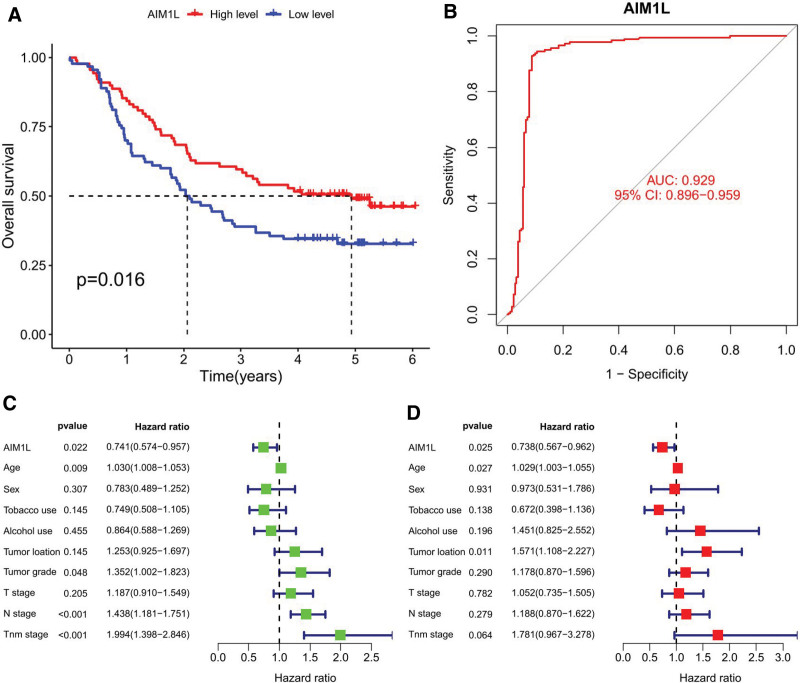
(A) OS curves of ESCC patients according to AIM1L. (B) ROC curve for AIM1L in GSE53625. (C) Univariate and (D) multivariate Cox regression analysis. AIM1L = absent in melanoma 1-like, ESCC = esophageal squamous cell carcinoma, OS = overall survival, ROC = receiver operating characteristic.

In GSE53625, 179 patients have comprehensive clinical information. According to the median AIM1L expression, 90 patients were classified into the low-expression group and 89 into the high-expression group. OS analysis showed that the low AIM1L expression group had a significantly lower survival rate than the high expression group (Fig. 2A). Furthermore, the ROC curve was plotted according to AIM1L expression values in GSE53625 with an area under the curve >0.9 (Fig. 2B), indicating that AIM1L has significant sensitivity and specificity in the diagnosis of ESCC. Cox regression analysis results showed that AIM1L is an independent prognostic factor of OS, with hazard ratio of 0.741 (95% CI, 0.574–0.957) and 0.738 (95% CI: 0.567–0.962), respectively (Fig. 2C and D).

### 3.3. Correlation between AIM1L and clinical characteristics

**Figure 3.**
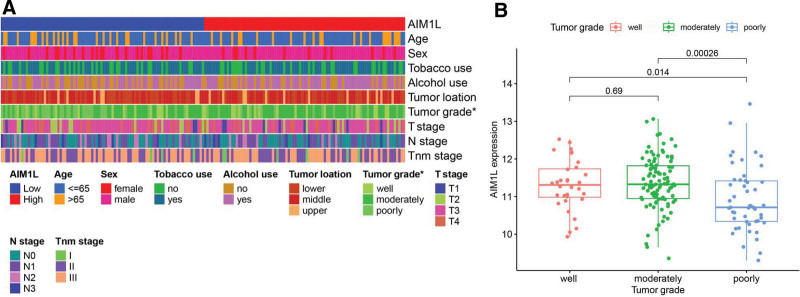
Association of AIM1L with clinical features. (A) Heatmap showing the distribution of clinical features between high and low AIM1L expression groups. (B) Differential expression of AIM1L for ESCC patients according to tumor grade. *P < .05. AIM1L = absent in melanoma 1-like, ESCC = esophageal squamous cell carcinoma.

We analyzed whether there is an association between AIM1L and the clinical features of ESCC patients, and the results showed that low expression of AIM1L generally indicates poor tumor grade in ESCC (Fig. 3A and B). However, we did not find any correlation of AIM1L with other clinical features.

### 3.4. Establishment and validation of a clinical prognostic model

**Figure 4.**
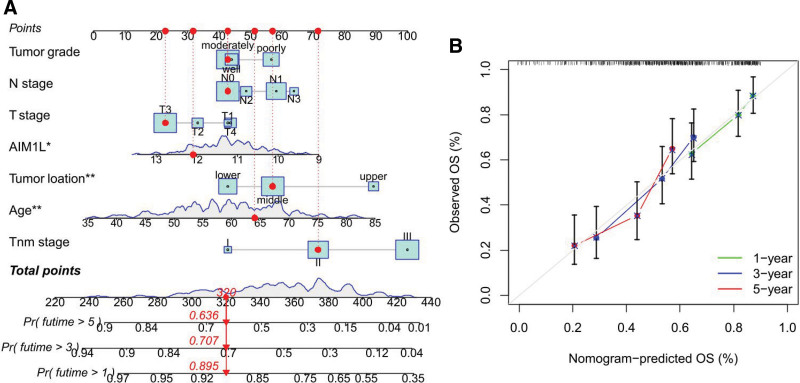
(A) Nomogram for OS prediction. (B) Calibration curves of the nomogram for 1-, 3-, and 5-year OS prediction. *P < .05, **P < .01. OS = overall survival.

We used the data in GSE53625 to construct a nomogram to predict the 1-, 3-, and 5-year survival rate of ESCC patients, AIM1L expression, age, tumor location, grade, T, N, and TNM stage were finally selected as parameters (Fig. 4A). The calibration curve showed good agreement between the nomogram predictions and the observed probability of survival for GSE53625 (Fig. 4B).

### 3.5. Identification of relevant genes for AIM1L and enrichment analysis

**Figure 5.**
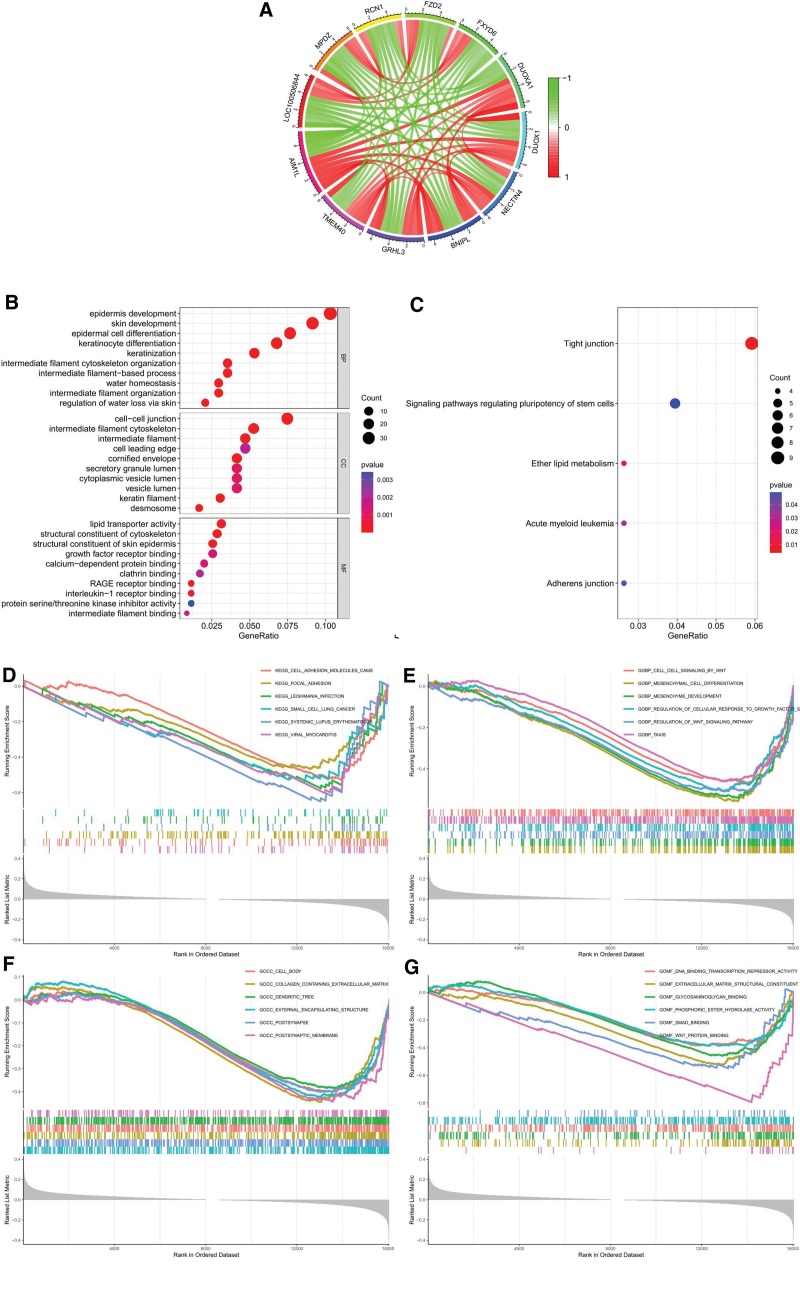
Identification of relevant genes for AIM1L and enrichment analysis. (A) Circos plot showing links between AIM1L and 11 genes in ESCC. (B) Results from GO analysis. (C) Results from KEGG enrichment analysis. (D–G) GSEA results in the low AIM1L expression group. AIM1L = absent in melanoma 1-like, ESCC = esophageal squamous cell carcinoma, GO = gene ontology, GSEA = gene set enrichment analysis, KEGG = Kyoto encyclopedia of genes and genomes.

Co-expression analysis based on GSE53625 found that 409 genes were significantly correlated with AIM1L, of which 113 genes were negatively correlated and 296 genes were positively correlated (Table S1, Supplemental Digital Content, http://links.lww.com/MD/J466). Figure 5A showed the association between AIM1L and 6 genes highly positively correlated with AIM1L and 5 genes highly negatively correlated with AIM1L. GO enrichment analysis showed that AIM1L-related genes are mainly enriched in epidermis development, skin development, epidermal cell differentiation in the biological process (BP), cell-cell junction, intermediate filament cytoskeleton, intermediate filament in the cell cycle, lipid transporter activity, structural constituent of cytoskeleton, and structural constituent of skin epidermis in the molecular function (MF) (Fig. 5B). In the KEGG project, they were mainly enriched in tight junction, signaling pathways regulating pluripotency of stem cells, ether lipid metabolism, acute myeloid leukemia, and adherens junction (Fig. 5C). GSEA showed that in the low expression group of AIM1L, viral myocarditis, cell adhesion molecules cams, focal adhesion were enriched in the KEGG project. Mesenchymal cell differentiation, mesenchyme development, regulation of Wnt signaling pathway were significantly enriched in BP. External encapsulating structure, postsynapse, collagen containing extracellular matrix were significantly enriched in cell cycle, and Wnt protein binding, extracellular matrix structural constituent were significantly enriched in MF (Fig. 5D–G, FDR < 0.05, NOM *P* < .05).

### 3.6. Relationship between AIM1L and immune cell infiltration in ESCC

**Figure 6.**
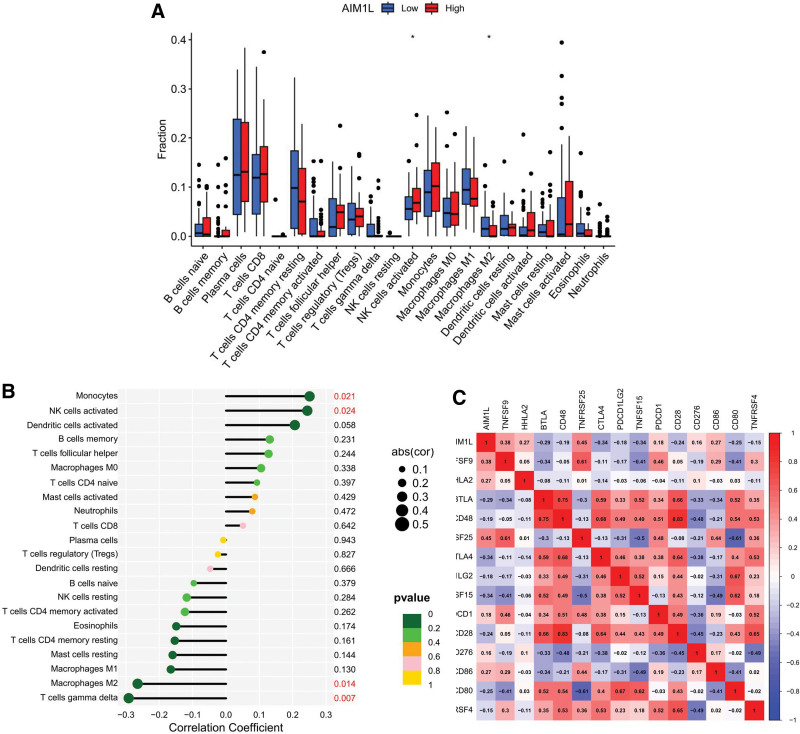
Relationship between AIM1L and immune infiltration. (A) Boxplot showing differences in AIM1L expression among 22 immune cells. (B) Lollipop plot showing a correlation between AIM1L expression and immune cell infiltration. (C) Correlation between AIM1L and immune checkpoint-related genes. *P < .05. AIM1L = absent in melanoma 1-like.

First, the immune infiltration information of 179 tumor samples obtained using the “CIBERSORT” package was filtered to obtain 85 meaningful samples (*P* < .05). After that, they were divided into 2 groups based on AIM1L expression, 43 cases into low expression groups. The results showed lower infiltration of activated NK cells in the low AIM1L expression group, whereas macrophage M2 was higher (Fig. 6A). According to Figure 6B, the infiltration level of activated monocytes and NK cells were positively correlated with AIM1L, while T cells gamma delta and macrophages M2 were negatively correlated with AIM1L. Figure 6C shows that AIM1L is positively correlated with immune checkpoint genes TNFRSF25, TNFRSF9, CD86, HHLA2, PDCD1, and CD276, and negatively correlated with TNFRSF15, CTLA4, BTLA, CD80, CD28, CD48, PDCD1LG2, and TNFRSF4 (*P* < .05).

### 3.7. Sensitivity analysis of AIM1L with anticancer drugs

**Figure 7.**
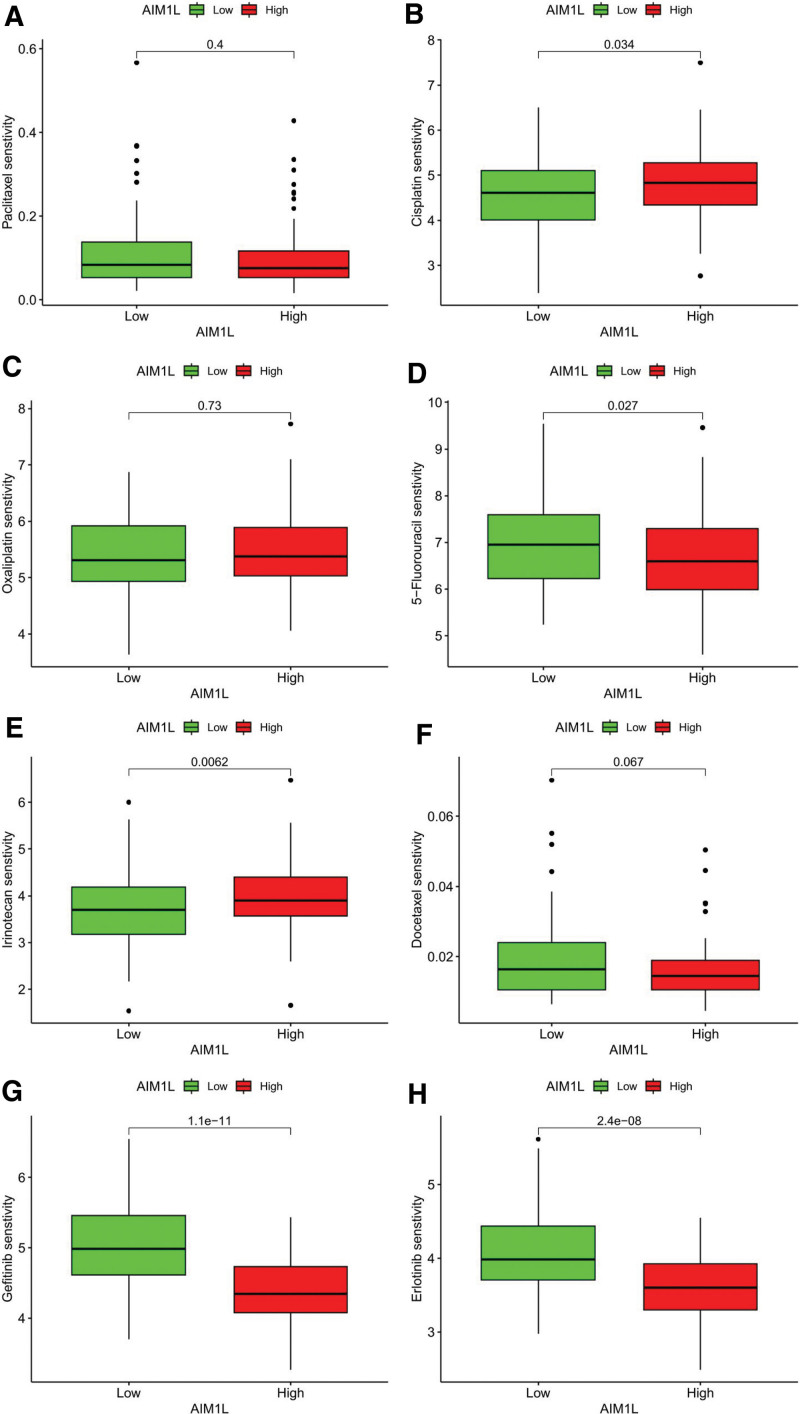
Therapeutic sensitivity prediction with AIM1L in ESCC. (A) Paclitaxel, (B) cisplatin, (C) oxaliplatin, (D) 5-fluorouracil, (E) irinotecan, (F) docetaxel, (G) gefitinib, (H) erlotinib. AIM1L = absent in melanoma 1-like, ESCC = esophageal squamous cell carcinoma.

We analyzed the differences in 50% maximum inhibitory concentration (IC50) of commonly used chemotherapeutic and molecularly targeted agents for ESCC treatment in AIM1L high and low expression groups. The results showed significantly higher IC50 for 5-fluorouracil, gefitinib, and erlotinib and lower IC50 for cisplatin and irinotecan in AIM1L low expression group. No significant association was found between AIM1L and paclitaxel, oxaliplatin, and docetaxel (Fig. 7).

### 3.8. Immunohistochemical staining

**Figure 8.**
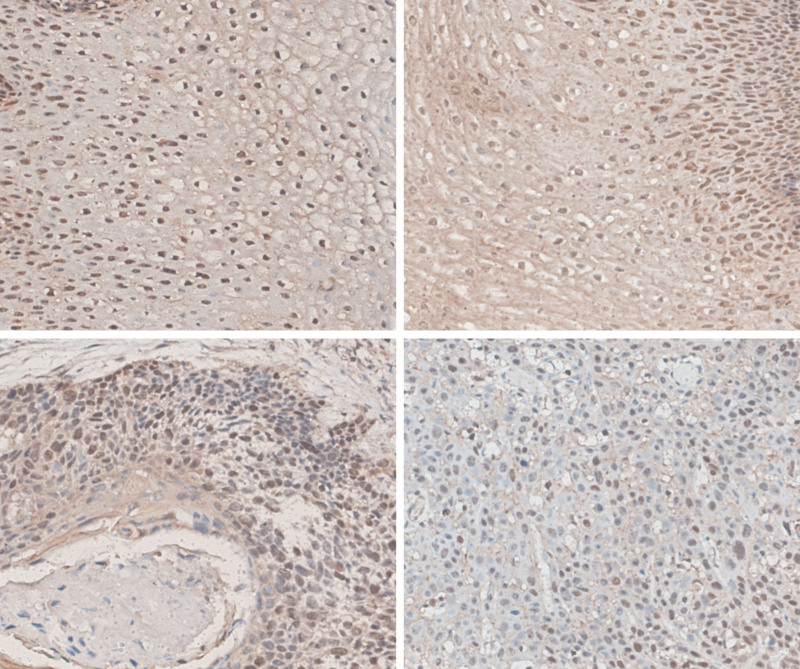
Immunohistochemical results. Compared to normal esophageal squamous epithelium, AIM1L is downregulated in the nucleus of esophageal squamous cell carcinoma. (A, B) Normal esophageal squamous epithelium. (C, D) Esophageal squamous cell carcinoma tissue. AIM1L = absent in melanoma 1-like.

According to our immunohistochemical results and chi-square test, we found that AIM1L was highly expressed in the cytoplasm of esophageal normal squamous epithelial tissues and cancer tissues (*P* > .05), whereas in the nucleus, AIM1L was highly expressed in esophageal normal squamous epithelial tissues (29/36) and cancer tissues (18/36), with significant difference (*P* < .05 Fig. 8).

## 4. Discussion

ESCC is one of the most lethal forms of human malignant tumors. It has the characteristics of late diagnosis, rapid metastasis, anti-treatment and frequent recurrence.^[19]^ Therefore, it is necessary to find new potential molecular markers of ESCC.

Based on our current research, we investigated whether AIM1L has a potential risk impact on ESCC. By mining and analyzing the gene expression data in the online database, we found that AIM1L mRNA in tumor tissue was down-regulated. In addition, in GSE53625, OS analysis, and Cox regression analysis showed that AIM1L was associated with poor prognosis of ESCC patients. ROC curve also showed that AIM1L has a high diagnostic value, and low AIM1L expression was also related to poor tumor grade. Immunohistochemistry revealed that AIM1L expression was downregulated in the nucleus of esophageal squamous cell carcinoma. These results suggest that AIM1L may be a potential diagnostic and prognostic marker for ESCC.

In addition, we found that in ESCC, TMEM40, GRHL3, BNIPL, NECTIN4, DUOX1, and DUOXA1 are highly positively correlated with AIM1L, while LOC100506844, MPDZ, RCN1, FZD2, and FXYD6 are highly negatively correlated with AIM1L, indicating that there may be a regulatory relationship between them. Previous studies have shown that GRHL3 can inhibit tumor growth and development in ESCC,^[20,21]^ while NECTIN4 and FZD2 play a role as cancer-promoting genes in ESCC,^[22,23]^ and no relevant report has been made on the role of other genes in ESCC. The specific relationship between AIM1L and these genes is still unclear, so it is necessary to explain the relationship between AIM1L and these genes in subsequent research. In MF of GO, AIM1L-related genes are enriched in growth factor receptor binding, RAGE receptor binding, interleukin-1 receptor binding, protein serine/threonine kinase inhibitor activity, etc. In KEGG analysis, these genes are enriched in acute myeloid leukemia, etc. GSEA showed that in the AIM1L low expression group, regulation of Wnt signal pathway and cell cell signaling by Wnt were enriched in BP, Wnt protein binding was enriched in MF. small cell lung cancer was enriched in KEGG. The Wnt signaling pathway plays an important role in the genesis and development of tumors,^[24]^ which indicates that the role of AIM1L in ESCC may be related to WNT signaling pathway, but further exploration is needed to clarify the relationship. The structure of AIM1L is similar to that of the absent in melanoma 1 (AIM1). Although there are few studies on AIM1L, AIM1 has been reported in various tumors. AIM1 hypermethylation is associated with bladder cancer, nasopharyngeal carcinoma, and melanoma.^[25,26]^ However, AIM1 is highly overexpressed in prostate cancer tissue.^[27]^ Another study on AIM1 and prostate cancer showed that advanced prostate cancer is usually accompanied by AIM1 deletion and decreased expression. The loss of AIM1 in prostate epithelial cells promotes cell migration and invasion. AIM1 can also inhibit the invasiveness of benign prostatic epithelium.^[28]^ AIM1 has also been identified as a potential tumor suppressor gene for malignant melanoma,^[29]^ and AIM1 hypermethylation is associated with poor prognosis of melanoma.^[30]^ Therefore, based on our research on AIM1L and related research, we cautiously assume that AIM1L may inhibit the occurrence and development of ESCC.

Chemotherapy, molecular targeted therapy, and immunotherapy hold a pivotal position in the treatment of ESCC.^[31]^ Therefore, we studied the correlation between AIM1L in ESCC and tumor-infiltrating immune cells, as well as some commonly used chemical drugs and molecularly targeted drugs. We found a positive correlation between AIML and activated NK cells, with a lower degree of infiltration in the low expression group of AIM1L, as opposed to macrophage M2. Previous studies have shown that NK cells achieve antitumor immunity through multiple routes of action, including cancer cells, stromal cell, and extracellular matrix, particularly metabolite interactions,^[32]^ while the high infiltration of macrophage M2 in ESCC can promote tumor progression.^[33]^ Our study showed that the higher the infiltration degree of activated NK cells, the better the prognostic value of ESCC patients, and macrophage M2 affected ESCC patients in contrast to activated NK cells, which is consistent with previous findings. On the other hand, drug sensitivity analysis showed that gefitinib, erlotinib, and 5-fluorouracil had lower treatment scores in the low AIM1L group, while cisplatin and irinotecan had higher treatment scores in the low AIML group. Epidermal growth factor receptor (EGFR) participates in a variety of cancer-related signal transduction pathways, and its tyrosine kinase activity plays a key role in mediating these processes.^[34]^ Gefitinib and erlotinib are EGFR tyrosine kinase inhibitors.^[31]^ Our results suggest that AIM1L may play a critical role in resistance to EGFR-targeted treatment in ESCC patients.

This study has its limitations. First, we acknowledge that most of this research is the mining and analysis of online databases, and only a few experiments have proved our speculation. Secondly, our sample size is not large enough. Therefore, we strongly recommend further research in this field to clarify the relationship between AIM1L and ESCC more clearly.

## 5. Conclusions

In conclusion, we studied the prognosis and biological role of AIM1L in ESCC. Low AIM1L expression is associated with poor prognosis and imbalance of tumor-infiltrating immune cells in ESCC. Patients with low AIM1L expression are more insensitive to EGFR inhibitors, thus affecting the prognosis of ESCC patients.

## Acknowledgments

We acknowledge our use of R software and GEPIA. The results are in part based upon data derived from GEO. We appreciate the platforms and the authors who uploaded their data.

## Author contributions

**Conceptualization:** Lu Zhou.

**Data curation:** Lu Zhou, Lanlan Gan.

**Formal analysis:** Lu Zhou, Lanlan Gan.

**Funding acquisition:** Lu Zhou.

**Investigation:** Lu Zhou, Lanlan Gan.

**Methodology:** Lu Zhou.

**Project administration:** Zongwen Liu, Lanlan Gan.

**Resources:** Lu Zhou.

**Software:** Lu Zhou.

**Supervision:** Zongwen Liu.

**Validation:** Lu Zhou.

**Visualization:** Lu Zhou.

**Writing – original draft:** Lu Zhou.

**Writing – review & editing:** Zongwen Liu.

## Supplementary Material


