# Supplementary material for: Expression and prognostic value of AIM1L in esophageal squamous cell carcinoma
Source: Medicine (Baltimore). 2023 Aug 25;102(34):e34677. doi: 10.1097/MD.0000000000034677 (PMC10470706; doi:10.1097/MD.0000000000034677)
Supplement: Supplementary file 1 [file medi-102-e34677-s001.pdf]

Table 1 Highly related gene with AIM1L in GSE53625			
Query	Gene	cor	pvalue
AIM1L	TMEM40	0.751719129	7.92E-34
AIM1L	GRHL3	0.733613099	1.64E-31
AIM1L	BNIPL	0.727116866	1.00E-30
AIM1L	NECTIN4	0.726706496	1.12E-30
AIM1L	DUOX1	0.724096399	2.28E-30
AIM1L	DUOXA1	0.7217106	4.34E-30
AIM1L	PERM1	0.717220151	1.43E-29
AIM1L	SFN	0.713681849	3.59E-29
AIM1L	LYPD3	0.711563303	6.20E-29
AIM1L	LINC01749	0.703983303	4.20E-28
AIM1L	PLEKHN1	0.700596475	9.69E-28
AIM1L	GPR45	0.699000746	1.43E-27
AIM1L	TGM1	0.698660332	1.55E-27
AIM1L	ITPKC	0.697565532	2.03E-27
AIM1L	ZBTB7B	0.690981626	9.75E-27
AIM1L	SLC4A1	0.690472644	1.10E-26
AIM1L	DUOXA2	0.689801751	1.29E-26
AIM1L	SBSN	0.689307481	1.44E-26
AIM1L	EPS8L1	0.689188347	1.49E-26
AIM1L	CCDC172	0.686990165	2.48E-26
AIM1L	KRT75	0.686510607	2.77E-26
AIM1L	TMPRSS11F	0.678956546	1.55E-25
AIM1L	MAST4	0.677852864	1.98E-25
AIM1L	LAD1	0.677179779	2.30E-25
AIM1L	KRT6C	0.669873847	1.15E-24
AIM1L	ZNF750	0.668273233	1.62E-24
AIM1L	RNF222	0.667802283	1.79E-24
AIM1L	CRYBB3	0.66644598	2.40E-24
AIM1L	ALS2CL	0.666323876	2.46E-24
AIM1L	RASAL1	0.665722617	2.79E-24
AIM1L	NCCRP1	0.656725179	1.84E-23
AIM1L	SPRR1B	0.655231366	2.50E-23
AIM1L	FAM83G	0.654806008	2.73E-23
AIM1L	KRT16	0.653274074	3.73E-23
AIM1L	MALL	0.652257717	4.58E-23
AIM1L	FAM46B	0.648879601	9.04E-23
AIM1L	C1orf116	0.648701114	9.36E-23
AIM1L	ZNF185	0.647686159	1.15E-22
AIM1L	SYTL1	0.647398591	1.21E-22
AIM1L	DEGS2	0.646354207	1.49E-22
AIM1L	MPZL2	0.643727887	2.51E-22

AIM1L	MXD1	0.642947664	2.92E-22
AIM1L	LINC01659	0.642937403	2.93E-22
AIM1L	SDC1	0.641645772	3.76E-22
AIM1L	TUBA4A	0.640781906	4.45E-22
AIM1L	LOC388282	0.640348809	4.84E-22
AIM1L	ZNF80	0.63895869	6.33E-22
AIM1L	LEXM	0.638123965	7.43E-22
AIM1L	GSDMC	0.636922082	9.36E-22
AIM1L	GIPC1	0.636740785	9.69E-22
AIM1L	S100A8	0.635911032	1.14E-21
AIM1L	LTB4R2	0.633793928	1.70E-21
AIM1L	CNKSR1	0.632723309	2.08E-21
AIM1L	CLEC18B	0.631394249	2.66E-21
AIM1L	CNFN	0.631032499	2.85E-21
AIM1L	FAM214B	0.630839029	2.96E-21
AIM1L	ACAP3	0.629200372	4.01E-21
AIM1L	DSG3	0.629071212	4.11E-21
AIM1L	PCDH1	0.628515712	4.56E-21
AIM1L	S100A14	0.627097302	5.92E-21
AIM1L	EPS8L2	0.626610688	6.48E-21
AIM1L	OVOL1	0.626460028	6.66E-21
AIM1L	TMEM184A	0.625207952	8.38E-21
AIM1L	RHOV	0.624931235	8.82E-21
AIM1L	PRSS22	0.624371304	9.77E-21
AIM1L	EVPLL	0.624129696	1.02E-20
AIM1L	TUBA3C	0.621289726	1.71E-20
AIM1L	GRHL1	0.620798835	1.87E-20
AIM1L	FAM25A	0.619677305	2.29E-20
AIM1L	C8orf49	0.618993404	2.59E-20
AIM1L	TTC22	0.61872409	2.71E-20
AIM1L	TMPRSS11D	0.618558283	2.79E-20
AIM1L	CLIC3	0.617999006	3.09E-20
AIM1L	PPL	0.617962227	3.11E-20
AIM1L	SPRR1A	0.614750501	5.50E-20
AIM1L	S100A9	0.613929707	6.35E-20
AIM1L	TMEM154	0.612909226	7.60E-20
AIM1L	VSIG10L	0.612337863	8.40E-20
AIM1L	CRCT1	0.608501167	1.64E-19
AIM1L	RAET1E	0.606079066	2.48E-19
AIM1L	KLK11	0.606035322	2.50E-19
AIM1L	SPRR2D	0.605290539	2.84E-19
AIM1L	IL1RN	0.604766901	3.11E-19
AIM1L	C16orf82	0.603128532	4.10E-19

AIM1L	PTK6	0.602102103	4.88E-19
AIM1L	GPATCH3	0.602001593	4.96E-19
AIM1L	CSTB	0.601515555	5.39E-19
AIM1L	SCEL	0.599737473	7.26E-19
AIM1L	CDA	0.599085421	8.10E-19
AIM1L	ARHGEF4	0.597437154	1.07E-18
AIM1L	GPR157	0.597359824	1.08E-18
AIM1L	HES5	0.596607647	1.22E-18
AIM1L	GHRHR	0.596555679	1.23E-18
AIM1L	FAM83A-AS1	0.596182279	1.31E-18
AIM1L	KRT5	0.595633891	1.44E-18
AIM1L	SPRR2A	0.595236788	1.53E-18
AIM1L	ARL4D	0.595086313	1.57E-18
AIM1L	MKNK2	0.594538202	1.72E-18
AIM1L	ESRP2	0.594503108	1.73E-18
AIM1L	S100A12	0.593970369	1.89E-18
AIM1L	TNP1	0.59198987	2.61E-18
AIM1L	LOC440117	0.591205938	2.97E-18
AIM1L	TGM5	0.590603668	3.27E-18
AIM1L	EPN3	0.589400432	3.97E-18
AIM1L	BARHL2	0.587953026	5.02E-18
AIM1L	IFFO2	0.586875185	5.96E-18
AIM1L	ALDH3B2	0.586512155	6.32E-18
AIM1L	MAP3K9	0.586255949	6.58E-18
AIM1L	P2RY2	0.585529862	7.39E-18
AIM1L	PPP1R13L	0.585226291	7.76E-18
AIM1L	USP12-AS2	0.584861238	8.22E-18
AIM1L	LTB4R	0.584486024	8.73E-18
AIM1L	KRT80	0.584193724	9.14E-18
AIM1L	UPK2	0.583292999	1.05E-17
AIM1L	SH3BGRL3	0.582726543	1.15E-17
AIM1L	NBL1	0.582344091	1.22E-17
AIM1L	CPTP	0.581954271	1.30E-17
AIM1L	JUP	0.581630154	1.37E-17
AIM1L	IL36G	0.581584729	1.38E-17
AIM1L	SH3PXD2A-AS1	0.577943864	2.44E-17
AIM1L	CRABP2	0.577434154	2.64E-17
AIM1L	LY6D	0.576818859	2.90E-17
AIM1L	GFOD1	0.575407313	3.61E-17
AIM1L	P2RX3	0.575100359	3.78E-17
AIM1L	ECM1	0.575087897	3.79E-17
AIM1L	S100A2	0.572226271	5.87E-17
AIM1L	GJB5	0.571660577	6.40E-17

AIM1L	ARHGAP27	0.570942114	7.14E-17
AIM1L	NLRX1	0.570233443	7.95E-17
AIM1L	KCNA7	0.569685696	8.63E-17
AIM1L	SLC4A9	0.569530743	8.84E-17
AIM1L	SDR9C7	0.569412803	9.00E-17
AIM1L	KRT14	0.56908112	9.46E-17
AIM1L	DHRS1	0.5684944	1.03E-16
AIM1L	DMKN	0.568034361	1.11E-16
AIM1L	ATP2B3	0.567769088	1.15E-16
AIM1L	OR4D2	0.567115762	1.27E-16
AIM1L	PDZK1IP1	0.566251172	1.45E-16
AIM1L	KCTD11	0.565579969	1.60E-16
AIM1L	GBP6	0.564987546	1.75E-16
AIM1L	IL20RB	0.56362254	2.14E-16
AIM1L	DENND2C	0.563471959	2.18E-16
AIM1L	MINK1	0.562934557	2.37E-16
AIM1L	C15orf62	0.562421975	2.55E-16
AIM1L	TOM1L2	0.562215414	2.63E-16
AIM1L	MFSD2B	0.562141251	2.66E-16
AIM1L	PGK1	0.561609367	2.88E-16
AIM1L	PATE3	0.561014508	3.14E-16
AIM1L	B3GNT8	0.560416832	3.42E-16
AIM1L	NOTO	0.560101709	3.59E-16
AIM1L	FGFR3	0.559348698	4.00E-16
AIM1L	EPHX3	0.558142724	4.77E-16
AIM1L	YOD1	0.557579334	5.18E-16
AIM1L	SLPI	0.557301458	5.39E-16
AIM1L	IVL	0.557166895	5.50E-16
AIM1L	IL36RN	0.557087382	5.56E-16
AIM1L	RDH13	0.5568218	5.78E-16
AIM1L	KRT6A	0.556738284	5.85E-16
AIM1L	MPIG6B	0.556707082	5.87E-16
AIM1L	ANKRD9	0.556168454	6.35E-16
AIM1L	PLA2G4E-AS1	0.555506978	6.98E-16
AIM1L	DEF6	0.554897768	7.62E-16
AIM1L	KRT6B	0.554740392	7.80E-16
AIM1L	KCNK7	0.553893177	8.80E-16
AIM1L	EPHA1	0.552490836	1.08E-15
AIM1L	DHCR24	0.549679948	1.60E-15
AIM1L	LINC01214	0.549074193	1.75E-15
AIM1L	VWA7	0.548893985	1.79E-15
AIM1L	NIPAL4	0.548834594	1.81E-15
AIM1L	PADI1	0.548532094	1.88E-15

AIM1L	TACSTD2	0.547679649	2.12E-15
AIM1L	PERP	0.547676886	2.12E-15
AIM1L	GPR62	0.547409365	2.21E-15
AIM1L	RIOK3	0.547368934	2.22E-15
AIM1L	ASCC2	0.547283772	2.24E-15
AIM1L	LINC00390	0.546714026	2.43E-15
AIM1L	PLEKHG5	0.546100251	2.65E-15
AIM1L	ADGRF4	0.544557596	3.28E-15
AIM1L	LOC100129931	0.544513637	3.30E-15
AIM1L	PRRG4	0.544434366	3.34E-15
AIM1L	TMEM63B	0.543720858	3.69E-15
AIM1L	OR10A6	0.543467022	3.82E-15
AIM1L	C2orf54	0.543182689	3.97E-15
AIM1L	LINC00856	0.543002909	4.07E-15
AIM1L	RNASE7	0.542412111	4.42E-15
AIM1L	STARD5	0.542397342	4.43E-15
AIM1L	GM2A	0.541638573	4.91E-15
AIM1L	KPRP	0.541325801	5.13E-15
AIM1L	PHLDB3	0.540838231	5.48E-15
AIM1L	SLURP1	0.540836015	5.49E-15
AIM1L	ABCA7	0.540204079	5.98E-15
AIM1L	RABGGTA	0.54005054	6.11E-15
AIM1L	MROH6	0.539884573	6.25E-15
AIM1L	KLK10	0.539625962	6.47E-15
AIM1L	LINC01957	0.535154098	1.19E-14
AIM1L	LYNX1	0.534830545	1.24E-14
AIM1L	KRTAP9-1	0.534799802	1.24E-14
AIM1L	CFAP65	0.534537722	1.29E-14
AIM1L	SCNN1B	0.534439693	1.31E-14
AIM1L	FABP5	0.534100553	1.37E-14
AIM1L	SSH3	0.5338843	1.41E-14
AIM1L	KDF1	0.533863889	1.41E-14
AIM1L	PI3	0.532982124	1.59E-14
AIM1L	SIRT7	0.532086867	1.79E-14
AIM1L	LY6G6C	0.532039766	1.80E-14
AIM1L	ABLIM1	0.531526034	1.93E-14
AIM1L	BSND	0.531324347	1.98E-14
AIM1L	SLC2A1	0.531097602	2.04E-14
AIM1L	LINC00917	0.530894388	2.10E-14
AIM1L	OR10C1	0.530659325	2.16E-14
AIM1L	GJB6	0.528044078	3.05E-14
AIM1L	FGF5	0.527982814	3.08E-14
AIM1L	MPO	0.527070993	3.47E-14

AIM1L	CST6	0.526771983	3.61E-14
AIM1L	B3GNT3	0.5266468	3.67E-14
AIM1L	FSCN2	0.526603462	3.69E-14
AIM1L	SERPINB13	0.52648746	3.75E-14
AIM1L	KRT17	0.526395676	3.79E-14
AIM1L	FGFBP1	0.525988093	4.00E-14
AIM1L	SLC39A2	0.525486069	4.27E-14
AIM1L	RDH12	0.525147924	4.46E-14
AIM1L	MLIP-IT1	0.524710083	4.72E-14
AIM1L	PIM1	0.524173917	5.06E-14
AIM1L	SLC27A4	0.524145661	5.08E-14
AIM1L	HCAR3	0.523956607	5.21E-14
AIM1L	CWH43	0.523330864	5.65E-14
AIM1L	SNX33	0.523027942	5.87E-14
AIM1L	ERN1	0.522751948	6.09E-14
AIM1L	PSD4	0.522614873	6.19E-14
AIM1L	SEMA4B	0.522389188	6.38E-14
AIM1L	ABHD11-AS1	0.522272731	6.47E-14
AIM1L	OR4S1	0.521406153	7.24E-14
AIM1L	GOLGA7B	0.521326853	7.31E-14
AIM1L	ARRDC1	0.52109988	7.53E-14
AIM1L	IL37	0.520845023	7.78E-14
AIM1L	GDPD3	0.520601333	8.03E-14
AIM1L	CTNNBIP1	0.520589239	8.04E-14
AIM1L	PLA2G4E	0.520185509	8.47E-14
AIM1L	LRRC72	0.519822156	8.87E-14
AIM1L	UNC13D	0.519573978	9.16E-14
AIM1L	RAD51D	0.519107265	9.72E-14
AIM1L	CFAP46	0.518775561	1.01E-13
AIM1L	FUT6	0.518399687	1.06E-13
AIM1L	LINC00854	0.518161359	1.10E-13
AIM1L	SLC10A6	0.517769524	1.15E-13
AIM1L	CDC42BPB	0.517765665	1.15E-13
AIM1L	PHOSPHO1	0.517720639	1.16E-13
AIM1L	GJB3	0.517655334	1.17E-13
AIM1L	AQP3	0.516288566	1.39E-13
AIM1L	LINC00592	0.516020456	1.44E-13
AIM1L	ZCCHC13	0.515052932	1.62E-13
AIM1L	LLGL2	0.514870025	1.66E-13
AIM1L	LIMK2	0.514710768	1.70E-13
AIM1L	BPIFC	0.514334525	1.78E-13
AIM1L	AHNAK2	0.514159678	1.82E-13
AIM1L	ABCG4	0.514150071	1.82E-13

AIM1L	SOWAHC	0.514000078	1.85E-13
AIM1L	CCDC85C	0.513957375	1.86E-13
AIM1L	FAM83C	0.513877082	1.88E-13
AIM1L	SH3GL1	0.512966109	2.11E-13
AIM1L	PKP1	0.512923351	2.12E-13
AIM1L	FAM151A	0.512712559	2.18E-13
AIM1L	NBEAL2	0.512492601	2.24E-13
AIM1L	MAB21L3	0.512052483	2.37E-13
AIM1L	LOC100128573	0.512038579	2.37E-13
AIM1L	EZR	0.511982377	2.39E-13
AIM1L	RARG	0.511954543	2.40E-13
AIM1L	CACNA1G	0.51194772	2.40E-13
AIM1L	CFAP73	0.511943431	2.40E-13
AIM1L	TNFRSF13C	0.511449365	2.55E-13
AIM1L	OR2Z1	0.511243289	2.62E-13
AIM1L	KLK9	0.509913605	3.09E-13
AIM1L	FAM163B	0.508536379	3.66E-13
AIM1L	TSHB	0.508508689	3.67E-13
AIM1L	TUBA8	0.508035402	3.89E-13
AIM1L	S100A7	0.507993021	3.91E-13
AIM1L	MIR210HG	0.50791102	3.95E-13
AIM1L	SERPINB3	0.507851853	3.98E-13
AIM1L	SLC22A23	0.50780471	4.01E-13
AIM1L	NUDT8	0.507630943	4.09E-13
AIM1L	SQRDL	0.507609099	4.10E-13
AIM1L	TICAM1	0.507338617	4.24E-13
AIM1L	LCE3E	0.507225867	4.30E-13
AIM1L	TNKS1BP1	0.506367862	4.78E-13
AIM1L	AFDN	0.504999757	5.65E-13
AIM1L	TDRD12	0.504966454	5.67E-13
AIM1L	KRTAP5-6	0.503798896	6.53E-13
AIM1L	PLD2	0.503514407	6.76E-13
AIM1L	ALOXE3	0.503471489	6.80E-13
AIM1L	BCL3	0.50228713	7.85E-13
AIM1L	LOC100507373	0.502054085	8.07E-13
AIM1L	SPINK5	0.50157909	8.55E-13
AIM1L	ARHGAP40	0.501296952	8.84E-13
AIM1L	ARHGEF18	0.500630948	9.58E-13
AIM1L	MAFK	0.500302715	9.97E-13
AIM1L	KRTAP29-1	0.500212918	1.01E-12
AIM1L	ANKRD6	-0.500690671	9.51E-13
AIM1L	ADGRG2	-0.501082285	9.07E-13
AIM1L	LCA5	-0.501173876	8.98E-13

AIM1L	TBC1D1	-0.501319001	8.82E-13
AIM1L	NFYB	-0.501762215	8.36E-13
AIM1L	SSR2	-0.503335137	6.91E-13
AIM1L	COA5	-0.503656314	6.65E-13
AIM1L	HDGFRP3	-0.504056363	6.33E-13
AIM1L	TNPO1	-0.504705428	5.85E-13
AIM1L	RBM12	-0.504981897	5.66E-13
AIM1L	FERMT2	-0.505377147	5.39E-13
AIM1L	USP51	-0.506264537	4.84E-13
AIM1L	RFXAP	-0.506370197	4.78E-13
AIM1L	ZEB1	-0.506497914	4.70E-13
AIM1L	EPC2	-0.506720365	4.58E-13
AIM1L	CYP2U1	-0.507823019	4.00E-13
AIM1L	VEZF1	-0.50890104	3.50E-13
AIM1L	PDE5A	-0.509024915	3.45E-13
AIM1L	ZBTB10	-0.509102233	3.41E-13
AIM1L	COL8A1	-0.509980301	3.06E-13
AIM1L	SHISA3	-0.510444241	2.89E-13
AIM1L	FAM19A5	-0.511361699	2.58E-13
AIM1L	SNTB1	-0.511401052	2.57E-13
AIM1L	STON1	-0.511460224	2.55E-13
AIM1L	CADM1	-0.511614746	2.50E-13
AIM1L	PKD2	-0.512626944	2.20E-13
AIM1L	MTR	-0.512747847	2.17E-13
AIM1L	ALG10B	-0.513329295	2.02E-13
AIM1L	LDLRAD4	-0.513353467	2.01E-13
AIM1L	PDE4B	-0.513700385	1.93E-13
AIM1L	S1PR3	-0.514987367	1.64E-13
AIM1L	SUSD5	-0.515340362	1.57E-13
AIM1L	SGCB	-0.515710817	1.50E-13
AIM1L	C16orf45	-0.518087063	1.11E-13
AIM1L	LOXL3	-0.518598551	1.04E-13
AIM1L	ZNF22	-0.518619246	1.03E-13
AIM1L	ABI2	-0.519558262	9.17E-14
AIM1L	RECK	-0.519827669	8.86E-14
AIM1L	SH2B3	-0.520695797	7.93E-14
AIM1L	CHPT1	-0.521124769	7.51E-14
AIM1L	MSRB3	-0.521587903	7.07E-14
AIM1L	SMAD9	-0.522970788	5.92E-14
AIM1L	CEP120	-0.52548308	4.27E-14
AIM1L	FKBP7	-0.525902687	4.04E-14
AIM1L	BACH2	-0.5260774	3.95E-14
AIM1L	RAB9B	-0.527641979	3.22E-14

AIM1L	MORN4	-0.528308301	2.95E-14
AIM1L	GNG2	-0.529637049	2.48E-14
AIM1L	SOCS5	-0.530059043	2.34E-14
AIM1L	CPQ	-0.530144097	2.32E-14
AIM1L	SHISA2	-0.530620379	2.17E-14
AIM1L	SPRED1	-0.530724925	2.14E-14
AIM1L	CGGBP1	-0.53088869	2.10E-14
AIM1L	PRKAR2B	-0.532468678	1.70E-14
AIM1L	SESTD1	-0.532599079	1.67E-14
AIM1L	TRAM2-AS1	-0.532738485	1.64E-14
AIM1L	APBA2	-0.533295821	1.52E-14
AIM1L	EPB41L2	-0.533387787	1.50E-14
AIM1L	MAP9	-0.534878786	1.23E-14
AIM1L	SYT11	-0.535223983	1.18E-14
AIM1L	TGIF2	-0.535893733	1.07E-14
AIM1L	ERLEC1	-0.538273501	7.78E-15
AIM1L	LEF1	-0.541476082	5.02E-15
AIM1L	NUDT6	-0.541570335	4.96E-15
AIM1L	EFNB3	-0.541619692	4.93E-15
AIM1L	B4GALT6	-0.544123752	3.49E-15
AIM1L	CENPV	-0.544202958	3.45E-15
AIM1L	RNF150	-0.544422056	3.35E-15
AIM1L	RGAG4	-0.544524993	3.30E-15
AIM1L	NAP1L3	-0.544806436	3.17E-15
AIM1L	HIP1	-0.544954998	3.11E-15
AIM1L	PKIG	-0.54522692	2.99E-15
AIM1L	SGCE	-0.546764693	2.41E-15
AIM1L	ZNF853	-0.547236419	2.26E-15
AIM1L	LIFR	-0.548595919	1.87E-15
AIM1L	AP1S2	-0.550707649	1.39E-15
AIM1L	EXTL2	-0.550726546	1.38E-15
AIM1L	NRXN2	-0.551230147	1.29E-15
AIM1L	ENOX1	-0.551366282	1.26E-15
AIM1L	TMEM55A	-0.551801152	1.19E-15
AIM1L	TMEM170B	-0.552251521	1.11E-15
AIM1L	GOLM1	-0.552668019	1.05E-15
AIM1L	MRPS14	-0.552787029	1.03E-15
AIM1L	EPDR1	-0.554785522	7.75E-16
AIM1L	CC2D2A	-0.555175155	7.33E-16
AIM1L	FZD1	-0.556942195	5.68E-16
AIM1L	TNS3	-0.557681499	5.10E-16
AIM1L	ANGEL2	-0.558365318	4.62E-16
AIM1L	JAM3	-0.562034262	2.70E-16

AIM1L	CEP170	-0.564099827	1.99E-16
AIM1L	ETV1	-0.564380186	1.91E-16
AIM1L	ANXA6	-0.564556028	1.86E-16
AIM1L	DPYSL2	-0.564939226	1.76E-16
AIM1L	RAMP2-AS1	-0.565974487	1.51E-16
AIM1L	TGOLN2	-0.571118967	6.95E-17
AIM1L	PDS5B	-0.572506147	5.63E-17
AIM1L	ATP11C	-0.57296611	5.25E-17
AIM1L	TMEM200A	-0.573196143	5.07E-17
AIM1L	KIF5C	-0.574971927	3.86E-17
AIM1L	FGFR1	-0.577153523	2.76E-17
AIM1L	ETV5	-0.58198664	1.30E-17
AIM1L	LAMC1	-0.584636306	8.52E-18
AIM1L	TIMP1	-0.584747844	8.37E-18
AIM1L	LDOC1L	-0.585274417	7.70E-18
AIM1L	PRKD1	-0.588510989	4.59E-18
AIM1L	TMEM98	-0.590059872	3.57E-18
AIM1L	SV2A	-0.590718838	3.21E-18
AIM1L	TUB	-0.595539945	1.46E-18
AIM1L	FXYD6	-0.601776264	5.16E-19
AIM1L	FZD2	-0.617020678	3.68E-20
AIM1L	RCN1	-0.618793752	2.68E-20
AIM1L	MPDZ	-0.674186096	4.47E-25
AIM1L	LOC100506844	-0.692004241	7.66E-27
